# Ontogenetic Tooth Reduction in *Stenopterygius quadriscissus* (Reptilia: Ichthyosauria): Negative Allometry, Changes in Growth Rate, and Early Senescence of the Dental Lamina

**DOI:** 10.1371/journal.pone.0141904

**Published:** 2015-11-18

**Authors:** Daniel G. Dick, Erin E. Maxwell

**Affiliations:** 1 Department of Paleontology, Staatliches Museum für Naturkunde Stuttgart, Stuttgart, Germany; 2 Department of Geoscience, Eberhard-Karls-Universität Tübingen, Tübingen, Germany; New York Institute of Technology College of Osteopathic Medicine, UNITED STATES

## Abstract

We explore the functional, developmental, and evolutionary processes which are argued to produce tooth reduction in the extinct marine reptile *Stenopterygius quadriscissus* (Reptilia: Ichthyosauria). We analyze the relationship between mandible growth and tooth size, shape, and count, to establish an ontogenetic trend. The pattern in *S*. *quadriscissus* is consistent with hypotheses of tooth size reduction by neutral selection, and this unusual morphology (a functionally edentulous rostrum) was produced by a series of different evolutionary developmental changes that are known for other taxa showing tooth reduction and loss. Specifically, this species evolved functional edentulism by evolutionary changes in the growth allometry of the dentition and by altering growth rates through ontogeny. This observation supports previous hypotheses that *S*. *quadriscissus* underwent ontogenetic tooth reduction. Tooth reduction in *S*. *quadriscissus* may be caused by unique selective pressures resulting from prey choice and feeding behavior, expanding our current understanding of the mechanisms producing tooth reduction.

## Introduction

Dentition has been convergently reduced in size or lost completely several times throughout vertebrate evolution, despite its importance in individual survival [1]. Loss of such a critical structure requires a complex interaction of developmental and environmental factors, and most extant species which show evolutionary “tooth reduction” are more accurately described as completely edentulous (and hence we use the term “tooth loss” to refer to this condition). Recent advances have improved our understanding of the processes resulting in either A) replacement of the dentition [1], or B) complete loss of the teeth [2]. However, our understanding of processes producing gradual tooth size reduction (without complete tooth loss) is comparatively limited. We demonstrate the existence of a previously hypothesized ontogenetic trend of increasing tooth reduction in the extinct marine reptile *Stenopterygius quadriscissus* (Reptilia: Ichthyosauria), and explore a potential mechanism producing gradual tooth reduction related to feeding behavior. In doing so, we expand our understanding of dental morphological evolution to include species which show functional edentulism without complete tooth loss.

### Tooth Reduction: Definitions and Hypotheses

In general, our understanding of the processes affecting dental development is restricted to species which show loss of mineralized tooth tissues [1] or complete loss of the dentition [2]. In these species the process of dental development is stopped or altered in early embryonic stages, before the onset of odontogenesis [2]. In addition to tooth loss of this type, some species show extreme reduction in the size of the teeth, resulting in what is functionally equivalent to tooth loss (as has been suggested for *Stenopterygius quadriscissus*) [3]. Complications in the literature have led to the term “tooth reduction” becoming problematic, as it is used indiscriminately to refer to a number of different morphological and developmental changes. For example, tooth reduction has been used to describe species which show; A) reduction in the size of the teeth relative to other species in the clade, B) species showing reduction in enamel structure, C) reduction in the number of teeth, due either to early senescence of the dental lamina or size reduction of the tooth bearing elements, and D) species which show A, B, and C in conjunction or some different combination [1,3–5]. For the purposes of this article, we define tooth reduction as a reduction in the size of the individual teeth, relative to other morphologically similar species in the same clade, or relative to an earlier ontogenetic stage. Tooth reduction can be further subdivided into a number of more specific terms; tooth height reduction, tooth crown height reduction, tooth tissue reduction, and functional tooth reduction. Here, we define tooth height reduction as a reduction of the entire exposed height of the tooth, including the crown height, the height of the ring of acellular cementum (where present), and the height of the exposed root. Tooth crown height reduction, which is the type of reduction discussed in this article, refers to reduction only of the height of the enamel crown. Tooth tissue reduction, which is not discussed here, can be defined as a reduction in the thickness, extent, and structure of specific tooth tissues, including the enamel, dentine, and cementum. Functional tooth reduction refers to situations wherein a combination of tooth height or tooth crown height reduction results in teeth too small to perform their ordinary function (specifically, teeth are considered functionally reduced if the teeth did not protrude beyond the dental groove, resulting in what is functionally equivalent to true edentulism). Finally, we use the term tooth count reduction to refer to reduction in the number of teeth.

### Genetic and Evolutionary Perspectives on Tooth Reduction and Loss

In addition to semantic confusion, using tooth reduction to mean different combinations of complete tooth or tissue reduction and loss has also created a problematic understanding of the mechanisms resulting in the different morphologies. Tooth loss is generally understood to be caused by changes in one or more odontogenic pathways which produce binary effects when altered (conventionally attributed to disruption of *Bmp4* or *Fgf8*, but this has been disputed recently [6]); switching these pathways off or prohibiting their interaction with antagonizing proteins results in a complete lack of dental development (and complete edentulism). Because of the clear effects of these changes, we have a relatively good understanding of the genetic and functional precursors necessary to produce complete tooth loss [5,7] (although see [6]). Specifically, in what we are calling the replacement hypothesis (*sensu* [1]), development of a novel morphology (i.e. the rhamphotheca) that functionally replaces the dentition allows for the teeth to be completely lost via one of these binary pathways. In most cases, the above mentioned mutations which result in a dramatic change in the structure of the teeth (or their complete loss) are fatal, due to the critical role played by the dentition in resource acquisition and processing [1]. In some cases, selective pressures on organisms to maintain a functioning dentition can be reduced due to a behavioral or morphological change [1,5]. To differentiate these adaptive or selectively neutral situations from aberrant cases of tooth reduction and loss (i.e. pathological or traumatic tooth loss), we use the term “evolutionary tooth reduction”. Evolutionary tooth reduction and loss occurs via a number of related mechanisms, all of which appear to involve down-tuning or shutting off of various protein pathways within the odontogenic mesenchyme and epithelium [2,8].

For example, in turtles it has been suggested that tooth loss is the product of mutations resulting in the impairment of *Shh* signaling in the oral epithelium causing termination of *Msx2* expression in the odontogenic mesenchyme, which in turn causes a failure of odontoblast development, producing complete edentulism [2]. Fossil evidence (i.e. *Proganochelys*) suggests that this tooth loss began rather rapidly in the marginal teeth, leaving early turtles with only palatal teeth, which were lost in a subsequent event [2]. The pathway resulting in edentulism in birds is different from that in turtles; limiting of *Bmp4* signaling in the oral epithelium, with a resultant loss of contact with the underlying odontogenic mesenchyme is proposed to halt odontogenesis ([9], but see [6]). Both cases represent a complete disruption of their respective pathways, which leads to failure of tooth development and resultant total dental agenesis. Given the binary effects of these mutations (i.e. teeth or no teeth), they are unlikely to explain the gradual evolutionary tooth reduction seen in many secondarily aquatic tetrapods.

Compared to the genetics of tooth loss, the genetic factors gradually leading to reduced tooth size (as defined above) are poorly understood. Plikus et al. [8] demonstrated that modifying the Bmp signaling pathway in the oral and dental epithelia of mice can result in marked changes in the dentition, including changes in the size of the individual teeth. Different from the conclusions of Harris et al. [9] (wherein the *Bmp4* pathway was completely shut off), Plikus et al. [8] demonstrated that tuning down the Bmp pathway (by increasing levels of the protein *noggin*) resulted in a decreased proliferation of odontoblasts (and consequently a smaller/deteriorated dentition). Where a reduced dentition is present in odontocetes, it is suggested to have evolved under similar conditions to those hypothesized above (specifically dental reduction due to alteration but not inactivation of the *Bmp4* pathway) [10]. However, in the odontocetes showing the greatest degree of tooth reduction (i.e. beaked whales), the advent of suction feeding plays a clear role in reducing the functional importance of the dentition [4], and consequently most beaked whale species have completely lost their teeth via the replacement method described above [1] (or had them extremely reduced to functional edentulism—i.e. *Tasmacetus shepherdi* [11]). As a result, despite morphological similarities, beaked whales and odontocetes are not a good model for the gradual tooth reduction seen in *S*. *quadriscissus*.

**Fig 1:**
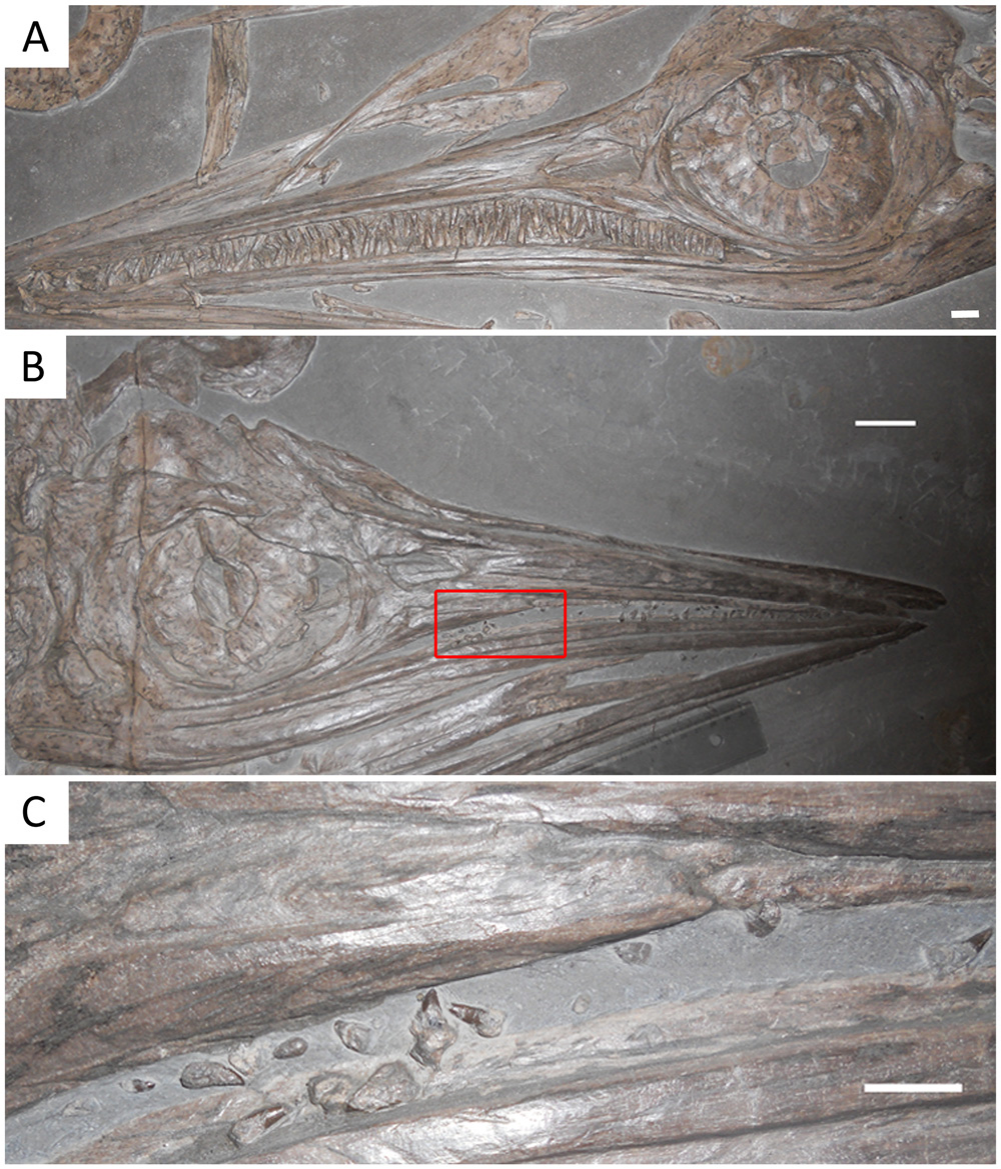
Image showing A) juvenile *Stenopterygius quadriscissus* (SMNS 54026) with relatively large, unreduced teeth (specimen mandible length: 368.67 mm). B) Large Adult *S*. *quadriscissus* (SMNS 53001), showing highly reduced, non-functional teeth (too short to protrude beyond the dental groove) (specimen mandible length: 523.2 mm). C) Close-up of the teeth seen in the box inset in B. Scale bars: A = 1 cm, B = 5 cm, and C = 1 cm.

Fossil ichthyosaurs of the genus *Stenopterygius* have been suggested to possess a highly reduced (in both size and number of teeth) or essentially absent dentition [12] (Fig 1). The teeth of *Stenopterygius* are described as reduced from two perspectives; firstly, they are reduced in relative size and structure when compared to closely related ichthyosaur species, such as *Ichthyosaurus communis* [9]. In *I*. *communis*, the teeth are relatively quite large and robust, possessing distinct ridges on the crown indicating a relatively thick enamel [12–13]. While the hypothesized tooth reduction in *S*. *quadriscissus* is apparent when considered interspecifically, it appears most distinct when viewed ontogenetically (Fig 1). Specifically, juvenile specimens of *S*. *quadriscissus* appear to possess more numerous and relatively larger tooth crowns; consequently, a hypothesis of ontogenetic tooth crown height (and tooth count) reduction has been suggested for this species in the literature [3]. Early descriptions of tooth reduction in *Stenopterygius* used tooth size as a taxonomic character, with highly reduced or completely absent teeth used to characterize *S*. *quadriscissus*, and unreduced teeth used to characterize species such as *S*. *megalorhinus*, among others [3]. However, these claims are problematic for a number of reasons, and tooth size is not considered a valid taxonomic character by many authors [14–15] (but see [12]). In a more recent taxonomic assessment [14–15], three species of *Stenopterygius* are recognized from the Early Jurassic. Intraspecific variation appears to exist in tooth size among adults of all species. Given the amount of intraspecific variation observed, it is unclear whether or not ontogenetic tooth reduction actually occurs in *S*. *quadriscissus*. In addition to these issues, debate has surrounded the argument regarding tooth count reduction, mainly based on issues of interpretation. Specifically, the unique tooth-attachment condition in ichthyosaurs has led to suggestions that the pattern of tooth count reduction seen may be a taphonomic artifact [16]. *Stenopterygius* is known to have a highly derived form of tooth attachment known as “aulocodonty” [17], wherein the teeth are set in a dental groove, but are not ankylosed to the bone [18]. As a result, it has been proposed that the “absent” dentition was in reality removed post-mortem by currents, scavenging, or some other process [16].

Tooth reduction has been proposed for ichthyosaur species other than *Stenopterygius* in the past. Sander *et al*. [16] suggested that the large ichthyosaur *Shastasaurus liangae* was completely edentulous, implying it was a suction feeder ecologically analogous to modern beaked whales (Ziphiidae). This suggestion was disputed by Motani *et al*. [19], who suggested that ichthyosaurs were morphologically incapable of being suction feeders. This complicates interpretations of tooth reduction in both *Stenopterygius* and *Shastasaurus*; as was described above, suction feeding can produce the necessary reduction in the function of the teeth necessary for their reduction and loss by gradual vestigial degeneration or by one of the binary mutations [16]. If ichthyosaurs such as *Shastasaurus* and *Stenopterygius* were incapable of suction feeding, then there appears to be no clear morphological or functional novelty capable of replacing the dentition (*sensu* [1]). Given this, convergent dental reduction in the absence of a functional replacement for the dentition could suggest that the teeth played a reduced role in feeding for many ichthyosaur species, allowing them to be lost by gradual vestigial degeneration. This possibility is explored in more detail below.

In this article we provide the first detailed exploration of ontogenetic tooth reduction in *Stenopterygius quadriscissus* (both in crown height and tooth count), with controls implemented to reduce the effects of taphonomic bias. We hypothesize that the extreme reduction of the teeth seen in *S*. *quadriscissus* resulted from a loss of selective pressure to maintain a functioning dentition due to the species’ feeding strategy, which allowed fixation of mutations resulting in; A) strong negative allometry, and B) early senescence of the successional dental lamina. We assess the morphological evolution of this species using a number of techniques from growth allometry, and discuss possible selective pressures that could produce a morphology similar to that seen in *S*. *quadriscissus*. This study improves our understanding of the evolutionary and developmental processes by which teeth can become reduced in size through ontogeny, as our current understanding restricts itself to evolutionary edentulism [1] or dental reduction by age-related mesenchymal stem cell senescence [20]. Additionally, we present a hypothetical example of a mechanism potentially capable of driving a gradual pattern of tooth size reduction.

## Materials and Methods

**Fig 2:**
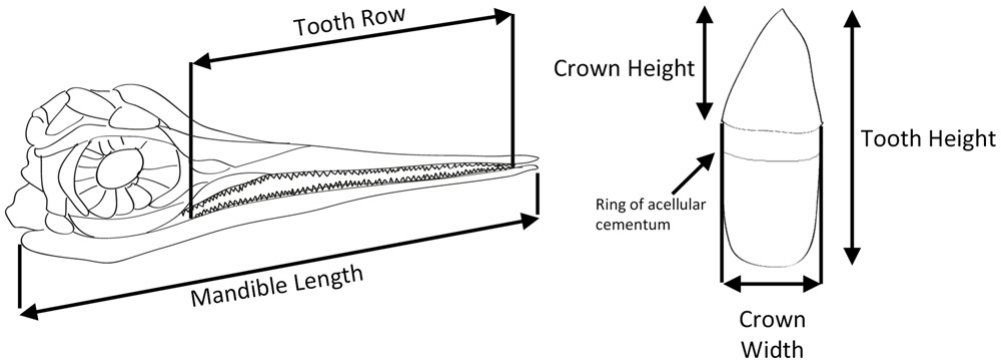
Diagram showing measurements used throughout the analysis.

Standardized measurements (crown height, crown base width, mandible length, and mandibular dental groove length) were taken from all teeth/skulls of *Stenopterygius* spp. specimens (n = 81) with known stratigraphy and definitive species identifications (based on the criteria in [15]) (Fig 2 and S1 Table). Specimens were measured from high-resolution photographs using AdobePro, and analyzed using PAST 3.06 [21]. Individual specimens ranged in size (and assumed age) from definitive embryos to large adults, and were classified into one of three species: *Stenopterygius quadriscissus* (n = 65), *S*. *triscissus* (n = 11) and *S*. *uniter* (n = 5). When assessing linear growth allometry, all measurements were log transformed prior to analysis (necessary to extract a signal that can be used comparatively; see [22]). A number of additional specimens initially measured could not be included in this analysis (n = 26) due either to their fragmentary nature, unclear taxonomic affinity, or missing/non-specific stratigraphic information. However, including/excluding these specimens did not affect the results. Only fully developed teeth were included, to avoid biasing the results by including the smaller-sized developing replacement teeth. All teeth measured in a single specimen were averaged, such that a single data point represents an individual specimen. To test the hypothesis of negative growth allometry described above, the resultant database was analyzed using Reduced Major Axis (RMA) regression, a standard technique for bivariate growth analysis [23]. While this analysis is standard for linear growth, analysis of residuals indicated an imperfect fit for the linear model (S1 Fig). Given this, the dataset was analyzed a second time, using a series of non-linear regression techniques [24], to attempt to determine the underlying biological relationship producing the observed pattern. Following the method described in [24], non-linear growth models were compared using Akaike’s information criterion (AIC), with the best model determined as the model showing a difference in AIC (ΔAIC) of greater than three (equivalent to a realized *p* value of 0.051; [24–25]) when present. Given that no model met these criteria for selection from the possible models (i.e. ΔAIC < 3 for all models considered—linear, von Bertalanffy, sigmoidal, and Gompertz, indicating no statistically significant difference), we elected to choose the von Bertalanffy growth model, as it had the highest AIC score, and can incorporate the possibility of asymptotic growth, which appears to occur (based on a visual inspection of the data) [24].

When assessing the linear relationship between the measurements included in this analysis, a species is said to grow with negative allometry when the slope of the RMA regression line is less than 1. Isometric growth occurs when the RMA slope is equal to 1 (or if the 95% confidence interval of the slope contains 1), and positive allometric growth is represented by slopes greater than 1. Following this analysis, specimens with complete (or mostly complete) dentitions were analyzed a second time, with their teeth divided into four quadrants (maxillary, premaxillary, anterior dentary, and posterior dentary), to assess any heterogeneity of growth within a single set of dentition, and to determine if there was systematic variation in tooth height throughout the dental arcade, which could potentially bias the results if a sampling of all dental quadrants was not available for all specimens. The null hypothesis of homogenous growth and tooth height between tooth quadrants was tested using One-way ANCOVA (with mandible length as a covariate). These same specimens were used to assess any possible reduction in total tooth count with increasing ontogenetic age. Finally, to test whether there was a corresponding change in tooth crown shape through growth, tooth shape (crown height/width) was compared with mandible length using RMA regression.

### Collections Abbreviations

SMNS = Staatliches Museum für Naturkunde Stuttgart, GPIT = Geologisches und Paläontologische Institut Tübingen, MMH = Urweltmuseum Hauff.

### Species Description and Geological Context

We use the taxonomy presented in [14] (see also [15]) for *Stenopterygius*, wherein three Toarcian species are recognized: *Stenopterygius quadriscissus*, *S*. *triscissus*, and *S*. *uniter*. *Stenopterygius* was a small to medium sized ichthyosaur, growing up to a maximum of around 3.5 meters in length [12]. Morphologically, *Stenopterygius* maintains the standard derived ichthyosaur body plan; thunniform and adapted for pelagic swimming [12]. The majority of specimens of *Stenopterygius* come from the Lower Jurassic (Toarcian) deposits of Germany, particularly the Posidonia Shale [15]. The Posidonia Shale was deposited approximately 180 million years ago in a shallow epicontinental sea known as the Southwest German Basin (SWGB), one of the many Central European epicontinental Basins [26]. The SWGB was characterized by a periodically dysoxic benthic environment, resulting in black shale deposition with high (>30%) total organic carbon (TOC) levels, and a limited benthic fauna [26]. Consequently, exceptionally preserved vertebrate fossils are known from this region, including complete, articulated skeletons (see for examples [27]). Given the exceptional nature of preservation of material within the Posidonia Shale, hypotheses suggesting that tooth reduction is an artifact created by taphonomic alteration [16] seem unlikely; small, easily dispersed bones such as distal phalanges and tail fluke vertebrae are frequently found in articulation, as are large numbers of teeth.

## Results

**Fig 3:**
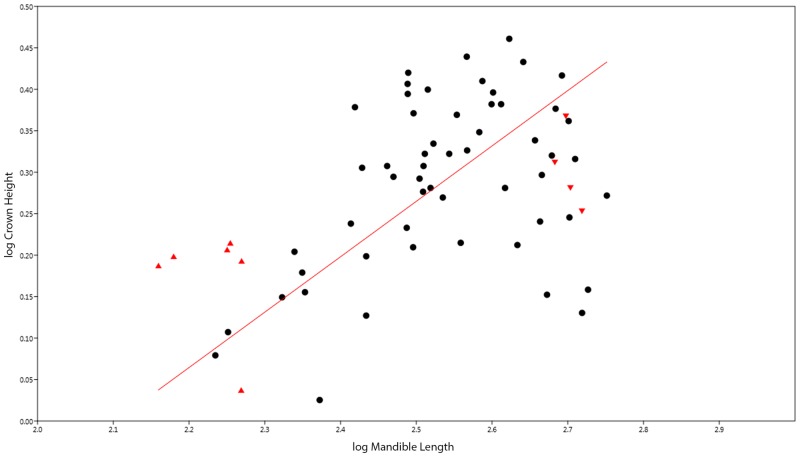
Graph showing RMA regression of logMandible vs. logCrownHeight. All values in log mm. Symbols: red-colored upward pointing triangles = embryos, red-colored downward pointing triangles = pregnant females.

The teeth of *Stenopterygius* are relatively simple; they are short, conical, and are essentially homogenous in shape throughout the dental arcade. An absence of macroscopic tooth wear could imply a minimized functional role for the teeth, or consistent consumption of soft-bodied prey. As predicted, *Stenopterygius quadriscissus* shows ontogenetic dental reduction, with small relative adult tooth size and clear negative allometry (Fig 3). The RMA slope *a* for logMandible against logCrownHeight for *S*. *quadriscissus* (Fig 3), with a value of 0.67 (95% CI of *a* = 0.53–0.79) shows strong negative allometry. This pattern holds for the RMA slope *a* for logMandibularToothRow against logCrownHeight as well (*a* = 0.74, 95% CI = 0.59–0.86). Because of the small sample sizes, a clear conclusion could not be reached for either *S*. *triscissus* or *S*. *uniter*, and the null hypothesis of isometry could not be rejected.

**Fig 4:**
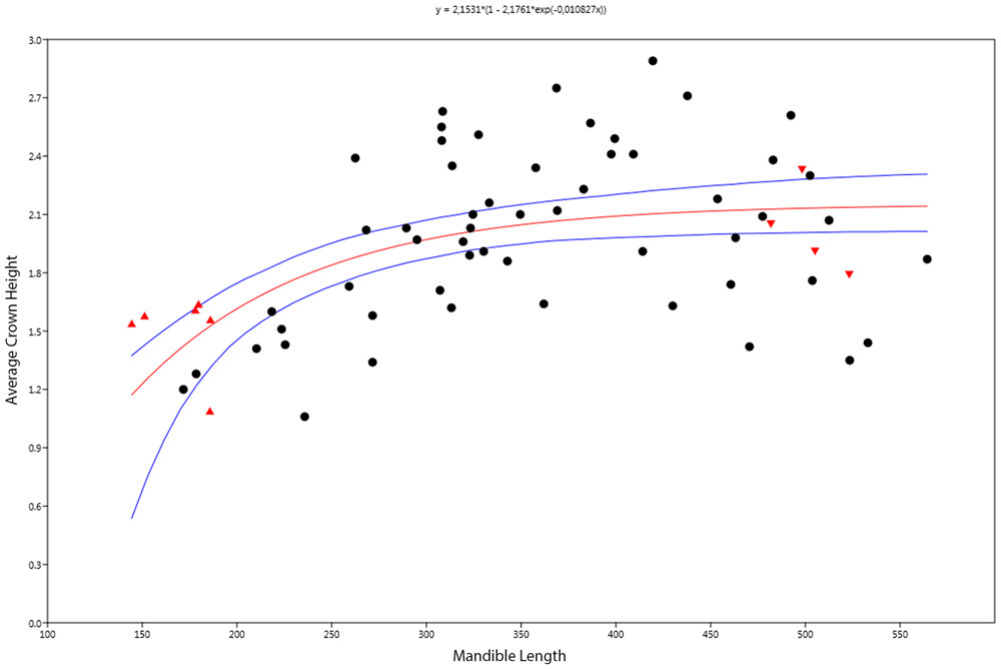
Dental growth in *S*. *quadriscissus* modelled using the von Bertalanffy growth model. Symbols: red-colored upward pointing triangles = embryos, red-colored downward pointing triangles = pregnant females. Values are raw measurements.

Fig 4 shows the results of fitting a von Bertalanffy growth model to the relationship between mandible length and crown height, which slightly improves the quality of the model (linear model AIC = 14.688, von Bertalanffy model AIC = 15.222). Tooth growth is initially slow, as was demonstrated above in the RMA analysis. The rate of dental growth appears to become asymptotic at mandible lengths greater than 350 mm (Fig 4). The high degree of intraspecific variation becomes apparent at mandible lengths greater than 400 mm, as there are adult specimens with absolutely smaller teeth than the juveniles [3]. The raw data suggest that relative tooth size is decreasing past this point; on average, absolute crown height increases between the juvenile (< 400 mm) and adult (> 400 mm = smallest pregnant female specimen) stages, but tooth size tends not to increase any further beyond mandible lengths of 420 mm (Fig 4).

**Fig 5:**
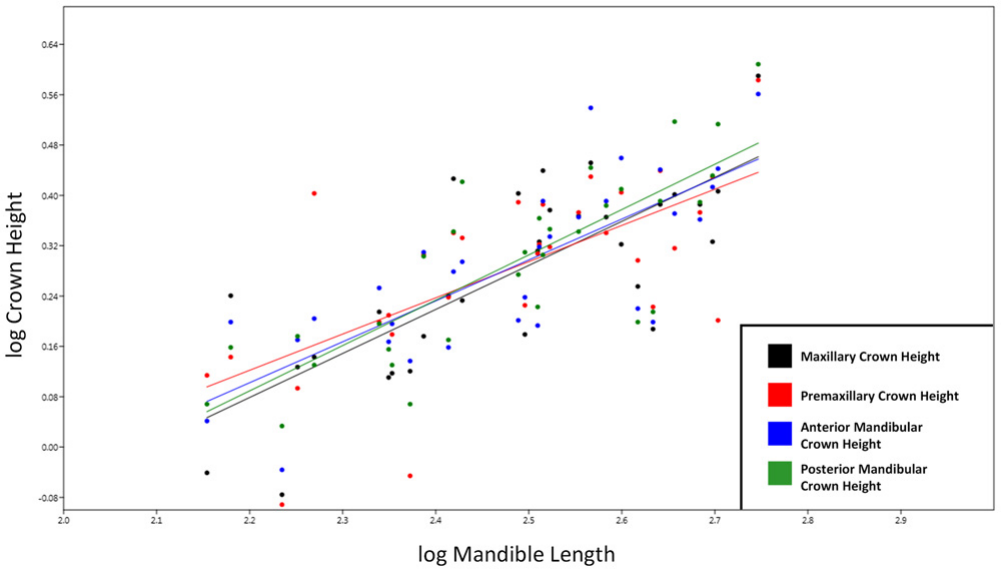
Plot showing the allometric slopes of the different tooth quadrants included in the One-way ANCOVA. Each vertical group of four points is a single specimen. Values = log mm.

The results of the One-way ANCOVA of the four dental quadrants in *S*. *quadriscissus* suggest that tooth reduction is homogenous throughout the dentition (Fig 5, S2 Table). While there appears visually to be substantial variation between quadrants at different ontogenetic stages, a statistically significant difference between the means could not be demonstrated (*p* = 0.928), and the null hypothesis of homogeneity of slope could likewise not be rejected (*p* = 0.7862). This implies there is no coordinated anterior-posterior variability within the dentition, and this seemingly random variation is discussed in more detail below.

**Fig 6:**
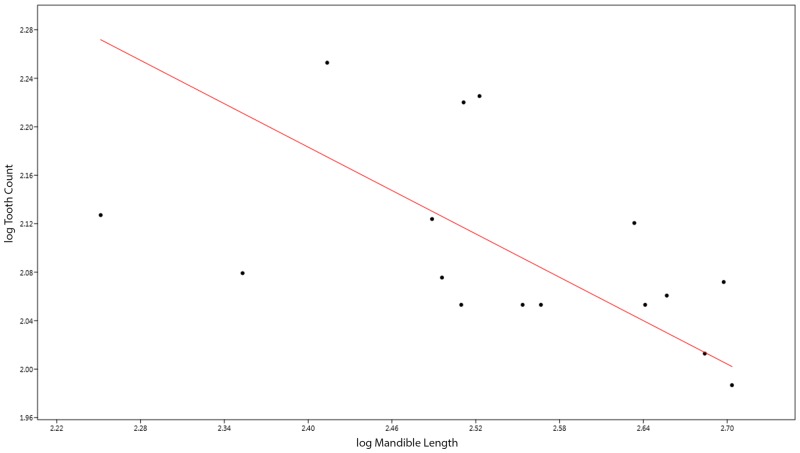
RMA analysis of tooth count through ontogeny in *Stenopterygius quadriscissus*.

The results of the tooth count reduction analysis indicated that there is a trend towards lower total tooth counts with increasing body size, and presumably ontogenetic age (logMandibleLength vs. logToothCount RMA slope *a* = -0.60, 95% CI of *a* = -0.91, -0.20) (Fig 6). In this analysis, the expected slope was zero or higher, based on the results of [28] demonstrating that in general tooth count increases through growth in reptiles. On average, juvenile specimens (n = 11) have approximately between 113 and 179 teeth preserved (mean: 137 teeth), and adults (n = 5) have between 97 and 119 teeth preserved (mean: 110 teeth). In the juvenile specimens, tooth count is greatest at the smallest mandible lengths included (i.e. SMNS 57009: mandible length 262.46 and 179 teeth present, SMNS 54026: mandible length 368.67 [largest juvenile with contiguous teeth] and 113 teeth). This pattern holds for adults as well. Because all specimens without a complete, contiguous dentition were excluded, the sample size was greatly reduced, and the reliability of this analysis is unclear. Additionally, it should be noted that the total tooth counts above represent one side of the dentition, as only one aspect is exposed in the specimens used; therefore the total mean bilateral counts would be approximately 274 teeth and 220 teeth for juveniles and adults, respectively. Of the few specimens available of *S*. *uniter* and *S*. *triscissus*, complete contiguous dentitions of the kind used in this analysis were uncommon. A large adult specimen of *S*. *uniter* (GPIT 1491/10) with a contiguous dentition had 242 teeth (total count—assuming equal distribution on both sides). A mid-sized juvenile specimen of *S*. *triscissus* was found with a total of approximately 266 teeth (GPIT 1297/1).

**Fig 7:**
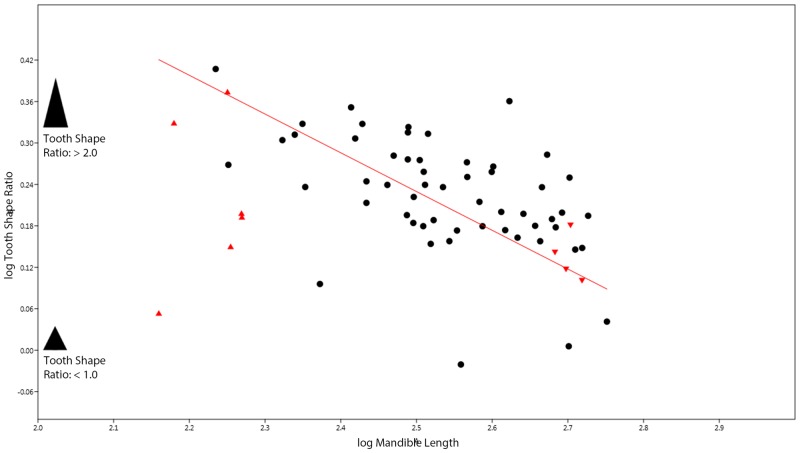
RMA analysis of tooth crown shape through ontogeny in *Stenopterygius quadriscissus*. Tooth Shape calculated as per Massare (1987).

Finally, the results of the RMA regression analysis assessing change in tooth shape suggest that the teeth in *S*. *quadriscissus* become increasingly short and robust through ontogeny (slope *a* = -0.56, 95% CI of *a* = -0.67, -0.43, expected slope = 0). (Fig 7).

## Discussion

Our results suggest that tooth reduction in *S*. *quadriscissus* occurs via a unique series of developmental changes, which allowed for extreme reduction in the size of the teeth (to the point that many specimens would have been functionally edentulous—i.e. teeth do not protrude beyond the dental groove) without complete loss of the teeth. Initially, negative allometric growth in early postnatal ontogeny (based on the simple linear relationship between tooth size and mandible length), followed by changes in growth rate at the point of sexual maturity significantly slowing the rate of growth, with a concomitant reduction in the number of teeth, potentially caused by early senescence of the dental lamina (see [29] for a review of the relationship between tooth count and the dental lamina). First, tooth size increases more slowly than jaw length in the earliest stages of postnatal ontogeny (Fig 3), and becomes increasingly slower towards maturity (Fig 4). Following this, the already small teeth stop increasing in size around sexual maturity (corresponding to a mandible length of around 400 mm, as seen in Fig 4 and discussed above). Continued mandibular growth beyond sexual maturity with negligible tooth growth causes the teeth to become relatively smaller, and to eventually become obscured by the dental groove or completely lost (Figs 3 and 4). Combined, this unusual growth pattern results in the extremely reduced, non-functional teeth seen in a number of specimens (see Fig 1).

Overall, this details one of the best examples of extensive tooth reduction without complete loss seen in any tetrapod species. Occurring concomitantly with these changes in growth rate and magnitude, there appears to be early senescence of the successional dental lamina (the tissue responsible for developing replacement teeth for those frequently lost), resulting in an increasingly lower tooth count following sexual maturity. This is an unusual pattern, as reptiles tend to increase the number of teeth as they grow [28]. Decreased *Bmi-1* expression through senescence can impact the ability of mesenchymal stem cells to differentiate into odontoblasts, resulting in decreased tooth count in the latest stages of life [19]. However, the potential onset of dental lamina senescence around maturity in *S*. *quadriscissus* is an unusual pattern, and further contributes to the extensive tooth reduction observed. Furthermore, that reduction in tooth count was demonstrated in specimens which clearly did not lose teeth post-mortem (i.e. a lower count where all teeth are *in-situ* and contiguous) suggests that other specimens with extremely low tooth counts (normally attributed to post-mortem loss–[16]) may in fact be accurate representations of the variation in adult tooth count in this species. Larger sample sizes are needed to increase confidence in this conclusion.

### Functional and Evolutionary Perspectives on Dental Reduction

Despite the morphological and genetic similarities, the reduced or absent teeth of different vertebrate species serve vastly different functional purposes. As described above, in most cases, tooth loss occurs following the evolution of a new morphological novelty to replace the dentition, which predisposes a species to tooth loss [1]. Interpreted in light of this “replacement hypothesis”, the ontogenetic tooth reduction seen in *Stenopterygius* appears unusual, as no morphological adaptation replaced the dentition. To explain this, here we suggest that the feeding strategy used by *Stenopterygius* produced the predisposition to tooth loss. Specifically, *Stenopterygius* has been previously described as a raptorial feeder within the “Smash” guild of marine reptiles (*sensu* [13]). Smash feeding involves a raptorial strike (rapid closing of the jaws) upon contact to crush or smash soft-bodied prey, which are then transported and swallowed [13,30–31]. The small, essentially non-functional teeth of adult *Stenopterygius* (particularly in specimens where the teeth do not protrude beyond the dental groove) seem to imply the force of closing the jaws was sufficient to stun or kill prey [13]. Furthermore, preliminary analysis of stomach contents suggests that even when feeding raptorially functionally edentulous specimens were capable of capturing prey [13]. Given that specimens we consider functionally edentulous (examples with teeth that do not protrude beyond the dental groove—i.e. SMNS 50187, SMNS 80115) have large agglomerations of belemnite hooklets preserved as stomach contents (pers. obs.), we propose that mutations resulting in smaller teeth would not have affected fitness in *Stenopterygius* (based on the only available evidence suggesting no difference in foraging success between functionally edentulous and “toothed” specimens). If the teeth served a reduced or non-existent function in feeding, mutations allowing for reduction in the size of the teeth could become fixed, due to the absence of stabilizing selection, resulting in slow vestigial degeneration (both the observed trend of negative allometry and the high adult variability observed support this suggestion, as a lack of stabilizing selection has been correlated with increased trait variability and vestigial degeneration–[32] and references therein).

**Fig 8:**
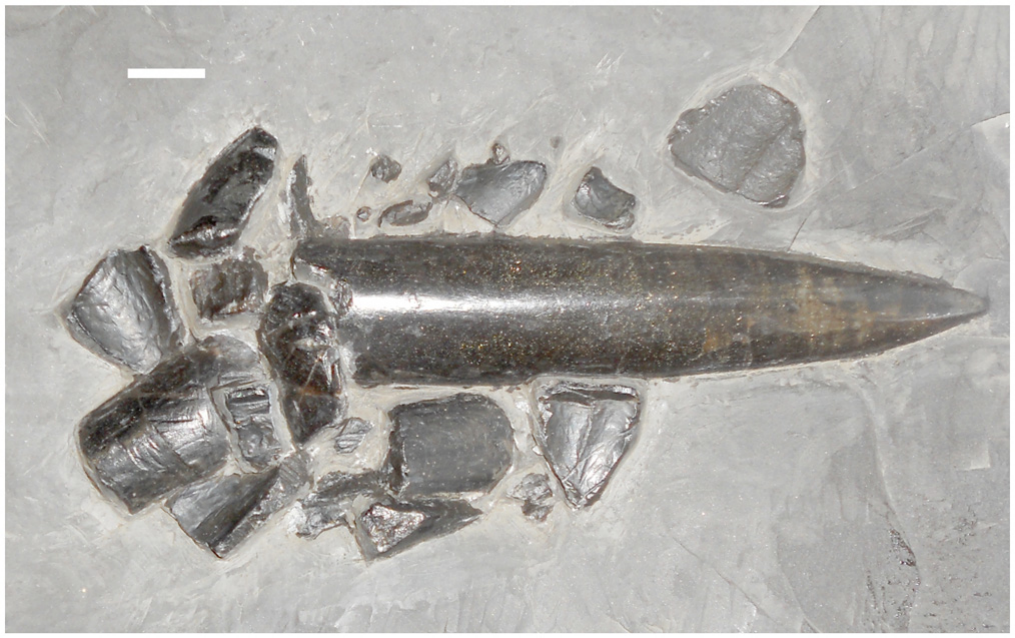
An example of a belemnite with a shattered phragmocone, interpreted as a bite mark (SMNS 60863). Scale bar = 1 cm.

Numerous examples of belemnites with shattered phragmocones from the Posidonia Shale (Fig 8) suggest some potential details into the feeding behavior of *Stenopterygius*, which could explain how smash feeding of the type described could allow the teeth to become subjected to neutral selection. Adult *Stenopterygius* appear to have been actively splitting the guards of belemnites from the softer body elements, and consuming the latter. This is supported both by the complete absence of stomach contents other than cephalopod hooklets [13, 33–36], and by the enormous number of isolated belemnite rostra found in the Posidonia Shale (and in particular by a number of examples showing a shattered phragmocone, hypothesized to have been caused by predation [37,38]) (Fig 8). As seen in Fig 8, precision bites to the weaker posterior aspect of the rostrum (the phragmocone, the proximal-most region with respect to the arms) appear to have been necessary to separate the soft tissue from the (presumably inedible) calcitic guard. Previous analyses of stomach contents support the suggestion that *Stenopterygius* avoided consuming the calcified rostra of belemnites; of the hundreds of *Stenopterygius* specimens studied, the only cephalopod remains present as gastric contents are belemnite hooklets (with not a single example containing a rostrum) [13,33–36]. This is contrary to the hypothesis advanced by [37], in which ichthyosaurs with reduced dentitions would have eaten primarily ‘naked’ cephalopods. Given that both functionally edentulous and specimens with teeth protruding beyond the dental groove have been found with belemnite hooklets preserved as stomach contents, the force of closing the jaws (or some other mechanism independent of tooth size) appears to have been sufficient to split the belemnites. Note that this does not imply smash feeding “causes” tooth reduction, merely that it allows for accumulation of mutations affecting the size and number of the teeth (by reducing the contribution of tooth size and shape to the overall fitness of the organism). This expands the “replacement hypothesis” [1], and suggests that particular feeding behaviors can reduce the fitness contribution of the dentition in the same way as morphological novelties (such as the rhamphotheca [1]).

## Conclusions

Our results demonstrate that, as previously predicted, *Stenopterygius quadriscissus* underwent ontogenetic tooth reduction. This reduction was produced via a unique and previously unrecognized combination of factors: negative growth allometry, ontogenetic changes in growth rate, and early senescence of the dental lamina. This produced dental reduction (and in extreme cases functional edentulism) in the absence of the binary (teeth or toothless) mutations commonly described in studies of tooth reduction and loss. Given that the teeth in *S*. *quadriscissus* were reduced to effectively functional edentulism in the absence of a morphological novelty to replace the dentition, the standard “replacement hypothesis” was unable to explain the observed reduction. We suggest that, when viewed from the perspective of its role in determining the function of the dentition, feeding specializations can replace the role of the dentition with regards to resource acquisition. In doing so, we expand the “replacement hypothesis” to include species which do not develop a morphological replacement for the dentition. This demonstrates a more unified outlook for understanding the processes resulting in tooth reduction and loss in tetrapods, and could potentially help to explain the unusually frequent convergence on tooth reduction/edentulism seen in secondarily aquatic fusiform predators.

## Supporting Information

S1 Fig Supplementary Fig 1. Residuals from the RMA analysis of log Mandible Length vs. log Average Crown Height.
(DOCX)

S1 Table Supplementary Table 1. All data used in this analysis. All values in mm, except “Tooth Count”, which is absolute number of teeth present on one side of the dentition.
(DOCX)

S2 Table Supplementary Table 2. Results of the One-way ANCOVA analysis described in the text.
(DOCX)
