# Supplementary material for: Ontogenetic Tooth Reduction in Stenopterygius quadriscissus (Reptilia: Ichthyosauria): Negative Allometry, Changes in Growth Rate, and Early Senescence of the Dental Lamina
Source: PLoS One. 2015 Nov 18;10(11):e0141904. doi: 10.1371/journal.pone.0141904 (PMC4651570; doi:10.1371/journal.pone.0141904)

**Supplementary Figure 1:** Residuals from the RMA analysis of log Mandible Length vs. log Average Crown Height.


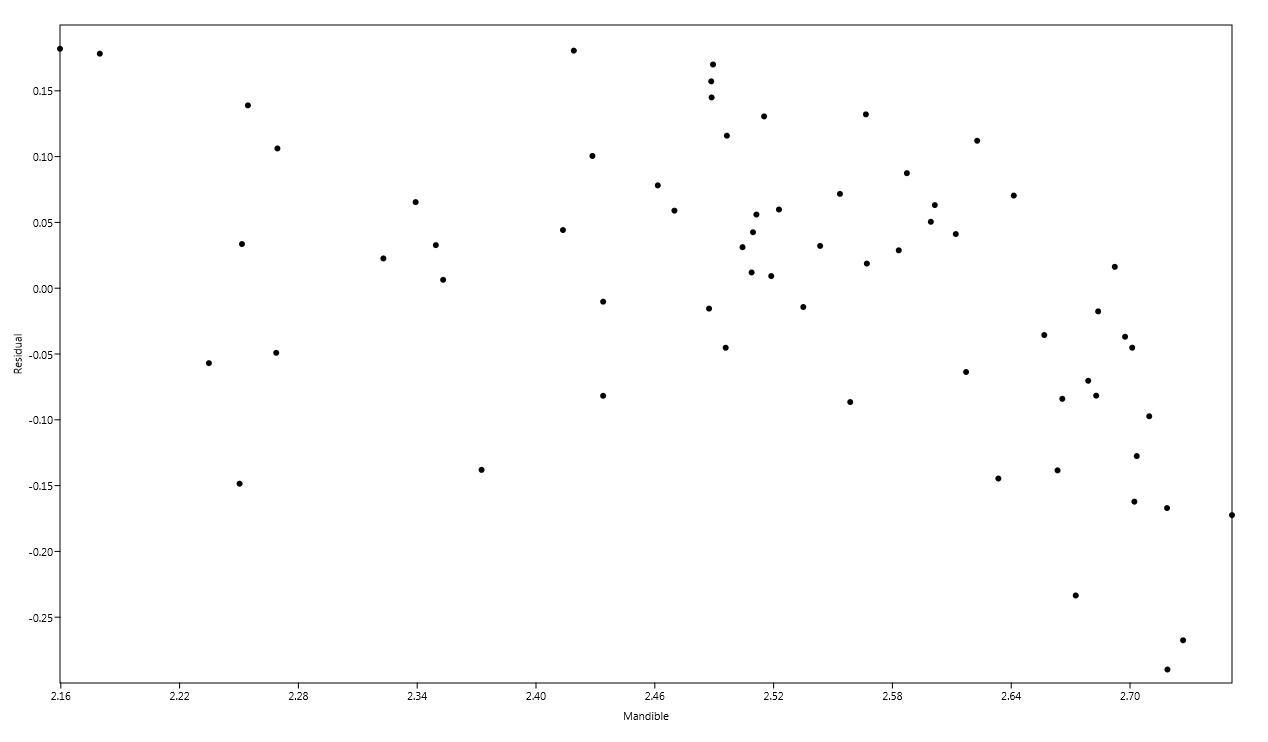

Supplement: S1 Fig — Residuals from the RMA analysis of log Mandible Length vs. log Average Crown Height. (DOCX) [file pone.0141904.s001.docx]
