# Supplementary material for: Ontogenetic Tooth Reduction in Stenopterygius quadriscissus (Reptilia: Ichthyosauria): Negative Allometry, Changes in Growth Rate, and Early Senescence of the Dental Lamina
Source: PLoS One. 2015 Nov 18;10(11):e0141904. doi: 10.1371/journal.pone.0141904 (PMC4651570; doi:10.1371/journal.pone.0141904)
Supplement: S1 Table — All data used in this analysis. All values in mm, except “Tooth Count”, which is absolute number of teeth present on one side of the dentition. (DOCX) [file pone.0141904.s002.docx]

**Supplementary Table 1:** All data used in this analysis. All values in mm, except “Tooth Count”, which is absolute number of teeth present on one side of the dentition.

| **Specimen #** | **Mandible Length** | **Avg. Crown Height** | **Avg. Crown Width** | **Tooth Row** | **Tooth Count** | **Species** |
| --- | --- | --- | --- | --- | --- | --- |
| SMNS 51133 | 460.69 | 1,74 | 1,21 | 278,93 | Incomplete | *S. quadriscissus* |
| SMNS 51142 | 502.5 | 2,3 | 2,27 | 342,06 | Incomplete | *S. quadriscissus* |
| SMNS 53001E | 179.69 | 1,64 | 1,16 | 160,38 | Incomplete | *S. quadriscissus* |
| SMNS 53001 | 523.2 | 1,79 | 1,42 | 353,66 | Incomplete | *S. quadriscissus* |
| SMNS 58276 | 327.52 | 2,51 | 1,22 | 266,17 | Incomplete | *S. quadriscissus* |
| SMNS 80062 | 369.09 | 2,12 | 1,19 | 276,5 | Incomplete | *S. quadriscissus* |
| GPIT 7298 | 397.57 | 2,41 | 1,33 | 263,12 | Incomplete | *S. quadriscissus* |
| SMNS 10418 | 307.98 | 2,55 | 1,35 | 225,72 | Incomplete | *S. quadriscissus* |
| SMNS 50003 | 323.29 | 2,03 | 1,12 | 224,8 | 113 | *S. quadriscissus* |
| SMNS 50165 | 503.73 | 1,76 | 0,99 | 338,14 | Incomplete | *S. quadriscissus* |
| SMNS 50963 | 409.25 | 2,41 | 1,52 | 311,92 | Incomplete | *S. quadriscissus* |
| SMNS 51101 | 271.59 | 1,58 | 0,9 | 211,25 | Incomplete | *S. quadriscissus* |
| SMNS 51140 | 223.57 | 1,51 | 0,71 | 159,71 | Incomplete | *S. quadriscissus* |
| SMNS 51554 | 437.79 | 2,71 | 1,72 | 284,21 | 113 | *S. quadriscissus* |
| SMNS 51828 | 178.44 | 1,28 | 0,69 | 157,93 | 134 | *S. quadriscissus* |
| GPIT 1491/9 | 564.35 | 1,87 | 1,7 | 437,99 | Incomplete | *S. quadriscissus* |
| SMNS 51947 | 399.42 | 2,49 | 1,35 | 268,9 | Incomplete | *S. quadriscissus* |
| SMNS 51948 | 512.53 | 2,07 | 1,48 | 276,3 | Incomplete | *S. quadriscissus* |
| SMNS 51959 | 235.76 | 1,06 | 0,85 | 163,09 | Incomplete | *S. quadriscissus* |
| SMNS 52531 | 333.17 | 2,16 | 1,4 | 241,53 | 168 | *S. quadriscissus* |
| SMNS 54026 | 368.67 | 2,75 | 1,47 | 254,21 | 113 | *S. quadriscissus* |
| SMNS 54051 | 324.56 | 2,1 | 1,21 | 222,88 | 166 | *S. quadriscissus* |
| SMNS 54064E | 144.41 | 1,54 | 1,36 | 128,94 | Incomplete | *S. quadriscissus* |
| SMNS 54816 | 470.54 | 1,42 | 0,74 | 338,81 | Incomplete | *S. quadriscissus* |
| SMNS 54818 | 218.38 | 1,6 | 0,78 | 129,66 | Incomplete | *S. quadriscissus* |
| SMNS 54819 | 308.57 | 2,63 | 1,25 | 235,46 | Incomplete | *S. quadriscissus* |
| SMNS 54872 | 268.22 | 2,02 | 0,95 | 187,66 | Incomplete | *S. quadriscissus* |
| SMNS 55109 | 225.46 | 1,43 | 0,83 | 167,33 | 120 | *S. quadriscissus* |
| SMNS 55343 | 533.03 | 1,44 | 0,92 | 391,05 | Incomplete | *S. quadriscissus* |
| SMNS 56615 | 308.11 | 2,48 | 1,2 | 216,9 | 133 | *S. quadriscissus* |
| SMNS 56631 | 319.39 | 1,96 | 1,04 | 225,29 | Incomplete | *S. quadriscissus* |
| SMNS 57009 | 259.20 | 1,73 | 0,77 | 186,52 | 179 | *S. quadriscissus* |
| SMNS 59706 | 289.43 | 2,03 | 1,17 | 222,03 | Incomplete | *S. quadriscissus* |
| SMNS 80113 | 453.67 | 2,18 | 1,44 | 313,84 | 115 | *S. quadriscissus* |
| SMNS 80115 | 463.2 | 1,98 | 1,15 | 303,86 | Incomplete | *S. quadriscissus* |
| SMNS 80226 | 414.17 | 1,91 | 1,28 | 231,79 | Incomplete | *S. quadriscissus* |
| SMNS 81367 | 386.59 | 2,57 | 1,7 | 284,38 | Incomplete | *S. quadriscissus* |
| SMNS 81962 | 357.72 | 2,34 | 1,57 | 233,44 | 113 | *S. quadriscissus* |
| SMNS 82046 | 262.46 | 2,39 | 1,18 | 180,94 | Incomplete | *S. quadriscissus* |
| SMNS 6293E | 185.7 | 1,09 | 0,69 | 128,74 | Incomplete | *S. quadriscissus* |
| SMNS 7402E | 185.95 | 1,56 | 1 | 127,59 | Incomplete | *S. quadriscissus* |
| SMNS 7402 | 498.29 | 2,33 | 1,78 | 344,85 | 118 | *S. quadriscissus* |
| SMNS 7800 | 313.20 | 1,62 | 1,06 | 200,94 | 119 | *S. quadriscissus* |
| SMNS 10460 | 477.47 | 2,09 | 1,35 | 344,89 | Incomplete | *S. quadriscissus* |
| SMNS 12821 | 171.70 | 1,2 | 0,47 | 112,69 | Incomplete | *S. quadriscissus* |
| SMNS 16811E | 151.24 | 1,58 | 0,74 | 141,81 | Incomplete | *S. quadriscissus* |
| SMNS 16811 | 505.13 | 1,91 | 1,26 | 303,13 | 97 | *S. quadriscissus* |
| SMNS 55934 | 430.04 | 1,63 | 1,12 | 282,57 | 132 | *S. quadriscissus* |
| SMNS 81958 | 349.53 | 2,1 | 1,46 | 265,26 | Incomplete | *S. quadriscissus* |
| GPIT 2799 | 383.00 | 2,23 | 1,36 | 240,3 | Incomplete | *S. quadriscissus* |
| GPIT U4E | 177.97 | 1,61 | 0,68 | 128,49 | Incomplete | *S. quadriscissus* |
| GPIT U4 | 481.82 | 2,05 | 1,48 | 300,3 | Incomplete | *S. quadriscissus* |
| SMNS 15033 | 482.99 | 2,38 | 1,58 | 311,5 | 103 | *S. quadriscissus* |
| SMNS 51471 | 361.97 | 1,64 | 1,72 | 241,65 | Incomplete | *S. quadriscissus* |
| SMNS 81841 | 307.16 | 1,71 | 1,09 | 219,26 | Incomplete | *S. quadriscissus* |
| GPIT U8 | 419.53 | 2,89 | 1,26 | 300,33 | Incomplete | *S. quadriscissus* |
| SMNS 56856 | 492.39 | 2,61 | 1,65 | 358,5 | Incomplete | *S. quadriscissus* |
| SMNS 81961 | 523.45 | 1,35 | 0,96 | 367,39 | Incomplete | *S. quadriscissus* |
| MMH 2A | 330.24 | 1,91 | 1,34 | 218,56 | Incomplete | *S. quadriscissus* |
| MMH 2B | 295.05 | 1,97 | 1,03 | 174,8 | Incomplete | *S. quadriscissus* |
| MMH 2D | 322.81 | 1,89 | 1,25 | 207,17 | Incomplete | *S. quadriscissus* |
| MMH 2E | 313.61 | 2,35 | 1,41 | 208,35 | Incomplete | *S. quadriscissus* |
| GPIT U9 | 342.78 | 1,86 | 1,08 | 228,52 | Incomplete | *S. quadriscissus* |
| MMH 2C | 271.57 | 1,34 | 0,82 | 188,02 | Incomplete | *S. quadriscissus* |
| MMH 2F | 210.32 | 1,41 | 0,7 | 155,94 | Incomplete | *S. quadriscissus* |
| SMNS 50376 | 631.83 | 6,87 | 2,98 | 430,54 | Incomplete | *S. uniter* |
| SMNS 57532 | 663.85 | 5,94 | 3,28 | 460,66 | Incomplete | *S. uniter* |
| GPIT 1491/10 | 557.97 | 3,84 | 1,86 | 359,47 | 121 | *S. uniter* |
| GPIT 1491/12 | 590.32 | 4,43 | 2,73 | 324,18 | 109 | *S. uniter* |
| SMNS 17500 | 660.15 | 3,02 | 1,88 | 374,96 | Incomplete | *S. uniter* |
| SMNS 54027 | 501.97 | 6,21 | 3,23 | 363,62 | Incomplete | *S. triscissus* |
| GPIT 5556 | 451.62 | 4,71 | 2,18 | 328,41 | Incomplete | *S. triscissus* |
| GPIT 7301 | 469.6 | 4,19 | 1,83 | 319,76 | 129 | *S. triscissus* |
| SMNS 50187 | 546.88 | 3 | 2,54 | 360,84 | Incomplete | *S. triscissus* |
| SMNS 55074 | 444.09 | 3,21 | 1,62 | 280,97 | Incomplete | *S. triscissus* |
| GPIT 9287E | 264.52 | 1,13 | 0,79 | 180,66 | Incomplete | *S. triscissus* |
| GPIT 29/9/7 | 320.48 | 1,54 | 1,02 | 199,87 | Incomplete | *S. triscissus* |
| GPIT 1297/1 | 337.52 | 1,46 | 1,18 | 214,39 | 133 | *S. triscissus* |
| GPIT U7 | 668.91 | 2,12 | 1,79 | 428,97 | Incomplete | *S. triscissus* |
| GPIT U7E | 229.16 | 1,36 | 0,92 | 157,8 | Incomplete | *S. triscissus* |
| SMNS 14846 | 646.76 | 3,14 | 2,04 | 366,04 | Incomplete | *S. triscissus* |
