# Supplementary material for: Ontogenetic Tooth Reduction in Stenopterygius quadriscissus (Reptilia: Ichthyosauria): Negative Allometry, Changes in Growth Rate, and Early Senescence of the Dental Lamina
Source: PLoS One. 2015 Nov 18;10(11):e0141904. doi: 10.1371/journal.pone.0141904 (PMC4651570; doi:10.1371/journal.pone.0141904)
Supplement: S2 Table — Results of the One-way ANCOVA analysis described in the text. (DOCX) [file pone.0141904.s003.docx]

**Supplementary Table 2:** Results of the One-way ANCOVA analysis described in the text.

| One-way ANCOVA results (Test for equal means, adjusted for mandible length). | | | | | |
| --- | --- | --- | --- | --- | --- |
|  | **Sum of sqrs** | **df** | **Mean square** | ***F*** | ***p* (same)** |
| **Adj. mean:** | 0.00415794 | 3 | 0.00138598 | 0.1524 | 0.928 |
| **Adj. error:** | 1.08237 | 119 | 0.00909555 |  |  |
| **Adj. total:** | 1.08643 | 122 |  |  |  |
| **Homogeneity (equality) of slopes:** | | | | | |
| ***F*:** | 0.3542 |  | | | |
| ***p* (same)** | 0.7862 |  |  |  |  |
